# MRI-DWI detection of residual cholesteatoma: moving toward an optimum follow-up scheme

**DOI:** 10.1007/s00405-024-08939-9

**Published:** 2024-09-13

**Authors:** Maura C. Eggink, Maarten J. F. de Wolf, Fenna A. Ebbens, Maartje M. L. de Win, Frederik G. Dikkers, Erik van Spronsen

**Affiliations:** 1 Department of Otorhinolaryngology, Head and Neck Surgery, Amsterdam University Medical Centre, Location University of Amsterdam, Amsterdam, The Netherlands; 2 Department of Radiology and Nuclear Medicine, Amsterdam University Medical Centre, Location University of Amsterdam, Amsterdam, The Netherlands; 3 Department of Otorhinolaryngology, Head and Neck Surgery, University Medical Centre Groningen, University of Groningen, Groningen, The Netherlands

**Keywords:** Cholesteatoma surgery, Follow up, Residual cholesteatoma, Non-EPI MRI DWI, Routine second-look surgeries

## Abstract

**Purpose:**

To analyse diagnostic accuracy of MRI-DWI in detecting residual disease after cholesteatoma surgery and propose an optimum follow-up (FU) scheme.

**Method:**

A retrospective chart review of patients who had cholesteatoma surgery in a tertiary referral centre. 3.0 T non-echo planar diffusion weighted imaging was performed as part of routine FU or indicated on the basis of clinical suspicion of disease. Imaging outcome was verified per-operatively during a second-look procedure or ossicular chain reconstruction. Diagnostic parameters were calculated and stratified by FU length.

**Results:**

For the FU of 664 cholesteatoma surgeries, 1208 MRI-DWI were obtained and 235 second-look procedures were performed. Most MRI-DWI were obtained within 1.5 yrs of surgery. In this period, significantly less true positive MRI-DWI and significantly more false negative MRI-DWI for residual disease were found compared to other FU periods. Scanning after approximately 3 yrs yielded a significantly higher rate of true positive MRI-DWI, while sensitivity surpassed 80%. Younger patients had a higher risk of developing residual disease. Patients undergoing canal wall up surgery, as well as patients < 12 yrs, were at risk for false negative MRI-DWI. Obliteration reduces the risk of residual disease, while leading to less false negative MRI-DWI.

**Conclusion:**

A novel radiologic FU scheme for detecting residual disease is suggested for stable ears after cholesteatoma surgery: standard MRI-DWI approximately 3 and 5 yrs after primary surgery, as well as MRI-DWI after approximately 9 yrs for patients with specific risk factors (i.e., patients < 12 yrs or patients undergoing canal wall up surgery without obliteration).

**Supplementary Information:**

The online version contains supplementary material available at 10.1007/s00405-024-08939-9.

## Introduction

Evolving surgical techniques in cholesteatoma surgery have necessitated complementary methods of follow-up (FU). Due to an increased risk of both recurrent and residual disease after canal wall up procedures compared to canal wall down procedures [1–3], second-look procedures, performed 9–12 months after primary surgery, was considered standard care for many years [4, 5]. Currently, an MRI scan with non-EPI DWI technique (non-echo planar imaging—diffusion weighted imaging, “MRI-DWI”), has widely replaced routine second-look procedures based on promising results of diagnostic accuracy in detecting residual disease after cholesteatoma surgery [6–11]. FU with imaging has several advantages over second-look procedures: it is non-invasive, cost-effective and allows for monitoring of the mastoid cavity, in cases of canal wall up surgery with obliteration [10, 12]. In our centre, MRI-DWI is performed at 1, 3 and 5 yrs after primary surgery as part of regular care. A second-look procedure is indicated when there is a clinical suspicion of residual or recurrent disease or when conductive hearing loss is present and a staged ossicular chain reconstruction (OCR) will be performed.

In medical literature, the reported sensitivity and specificity for MRI-DWI in predicting cholesteatoma recidivism (i.e., the presence of either recurrent or residual disease, or both) is highly variable, ranging from 40–100% and 50–100%, respectively [5, 7, 10, 13–24]. To date, no worldwide consensus has been reached regarding an optimum scheme of FU using MRI-DWI after cholesteatoma surgery [13]. Specifically, the ideal interval between primary surgery and first MRI-DWI, as well as intervals between the successive scans and total length of FU, have not yet been determined. The question also remains whether a specific protocol is necessary for patient groups with increased risk of residual disease, such as children and patients who underwent canal wall up surgery [1–3, 25].

In the Netherlands, a guideline on radiologic FU after cholesteatoma surgery was published in 2020 [26]. It proposed MRI-DWI at 1, 3 and 5 yrs post-operatively, while scans at 1, 2, 4 and 6 yrs were advised for paediatric patients. This advice however, was mostly based on consensus as studies on timing of MRI-DWI were lacking. The objective of this study is to evaluate our standard care pathway by analysing the diagnostic parameters of MRI-DWI at multiple points in time, in order to suggest improvements to the current FU scheme.

## Materials and methods

### Participants

All patients who underwent cholesteatoma surgery between January 1st 2004 and February 1st 2021 in the Amsterdam University Medical Centre, location University of Amsterdam, were reviewed. Institutional Review Board (IRB) approval was waived by the Medical Ethics Review Committee of the Amsterdam University Medical Centre. Patients with primary cholesteatoma were included, as well as patients referred to our tertiary referral centre after earlier cholesteatoma surgery. Surgical procedures were grouped in techniques with or without obliteration after mastoidectomy, as well as techniques leaving the canal-wall intact following mastoidectomy or not. As FU, MRI-DWI were obtained as part of routine FU after approximately 1, 3 and 5 yrs, or indicated on basis of clinical suspicion of disease. Six FU periods were defined: < 1.5 yrs; approximately 2 yrs, 3 yrs, 4 yrs, 5 yrs and > 5.5 yrs after initial surgery. A second-look procedure was performed on indication: for OCR; in case of doubt of complete removal of cholesteatoma; on clinical suspicion of recidivism or following a positive MRI-DWI. Residual disease was defined as growth of cholesteatoma in the previously described cholesteatoma localization as a result of incomplete removal [27]. Recurrent disease was defined as the reformation of a cholesteatoma from a novel retraction pocket [27]. Recidivism was be defined as the sum of both residual and recurrent disease. A retraction pocket without any keratin accumulation was regarded negative for cholesteatoma. Patients were excluded when the proposed second-look procedure was refused or when MRI-DWI was not available.

### MRI-DWI protocol, imaging evaluation

All patients were scanned on 3.0T MR systems (Ingenia and Ingenia Elition, Philips Medical Systems International B.V.). The MRI protocol consisted of an axial turbo spin-echo T2-weighted imaging (WI) and a coronal tse T1WI, with slice thickness of 3 mm. The non-echo planar DWI was acquired with a tse single shot technique with fat suppression in the axial and coronal direction, with slice thickness of 3 mm and b-value of 0 and 1000 s/mm^2^ (2012–2014) or 1500 s/mm^2^ (2015 onwards). From the DWI, apparent diffusion coefficient maps were calculated. In the rare case of a contra-indication for 3T, such as a pace-maker, a patient was scanned on 1.5T MR system. Scans obtained before the introduction of non-echo planar DWI (1st of January 2012), were excluded.

MRI-DWI was positive for cholesteatoma when there was a hyperintense lesion on the b1500, corresponding with a low value on the ADC map, a hypointense lesion on T1WI and hyperintense lesion on T2WI.

Imaging assessment was performed as part of clinical routine by a group of radiologists specialized in head- and neck and neuroradiology. If the scan was deemed uninterpretable by a radiologist due to technical aspects such as artefacts, it was excluded. The outcome of MRI-DWI was extracted from the radiological report and was dichotomized to be “positive” or “negative”.

Positive MRI-DWI were regarded “true positive” (TP) when following surgery revealed residual cholesteatoma or “false positive” (FP) when no cholesteatoma was objectified. When successive scans were marked positive but no cholesteatoma was found per-operatively, all prior scans were marked FP. Negative MRI-DWI were marked “true negative” (TN) when no cholesteatoma was found per-operatively or “false negative” (FN) when surgery did reveal residual cholesteatoma.

### Statistical analysis

Statistical analyses were performed in SPSS 28.0 (Chicago, IL, USA). Non-parametric tests were used to identify correlations between MRI-DWI findings and various factors. For these analyses, MRI-DWI were excluded in case of deliberate per-operative macroscopic residual disease, including subsequent FU scans (“known disease”). A significance level of *p* < 0.05 was used.

**Fig. 1:**
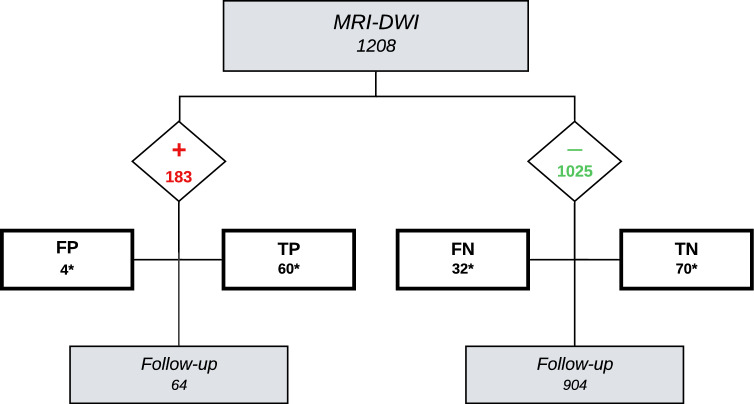
Flowchart showing possible outcomes of 1208 MRI-DWI performed in 555 ears. Numbers indicate amount of MRI-DWI. + : scan marked positive for residual or recurrent disease; − : scan marked negative for residual or recurrent disease. *TP* true positive for residual disease; *TN* true negative for residual disease; *FP* false positive for residual disease; *FN* false negative for residual disease. Values with asterisk (*) were used for calculating diagnostic parameters of MRI-DWI detecting residual disease. Please note 55 positive scans were found to be true positive for recurrence and 19 negative scans were found to be false negative for recurrence (not shown)

Diagnostic parameters were calculated (using values with asterisk in Fig. 1 and data in supplemental Table 1).

**Table 1:** Clinicopathologic characteristics of 555 ears who underwent surgery for cholesteatoma

Ears *n* = *555*	Median years (interquartile range)
Age of primary surgery	32.0 (35)
Radiologic follow-up	2.6 (3.4)
Surgical follow-up	2.7 (3.4)

## Results

### Research population

Retrospective database review yielded 491 patients corresponding with 555 ears, as 64 patients had bilateral cholesteatoma. Median age at initial surgery was 32.0 yrs (Table 1). In total 664 surgeries were performed by 12 otologic surgeons; canal wall up with obliteration being the most frequent (Table 2). As FU, 1309 MRI-DWI were obtained, of which 1208 after the introduction of non-echo planar DWI in 2012. Median length of radiologic FU was 2.6 yrs, with a maximum of 31.6 yrs after surgery. This wide range can be explained by radiologic FU of a contralateral cholesteatoma. In parallel, 235 second-look procedures were performed at a median of 2.7 yrs after initial surgery, with a maximum of 28.1 yrs.

### MRI-DWI findings

MRI-DWI findings are presented in Fig. 1, supplemental table 1 and supplemental Fig. 1. In total, the results of 166 MRI-DWI could be verified retrospectively by the per-operative findings of a second-look surgery performed. Four ears had FP MRI-DWI: one case noted a previously placed Silastic™ sheet, two cases had ear wax accumulation in a previously operated external ear canal (supplemental Fig. 1) and one case had no unusual findings. Sixty-four patients with positive scans are still in FU: five are clinically suspected to be positive for residual disease and eight for recurrent disease (all awaiting surgery) and 51 were deemed positive but “wait-and-scan” was chosen. Numerous negative scans (904) have not been verified by surgery and are still in clinical FU (Fig. 1).

### MRI-DWI findings stratified by period of FU

**Fig. 2:**
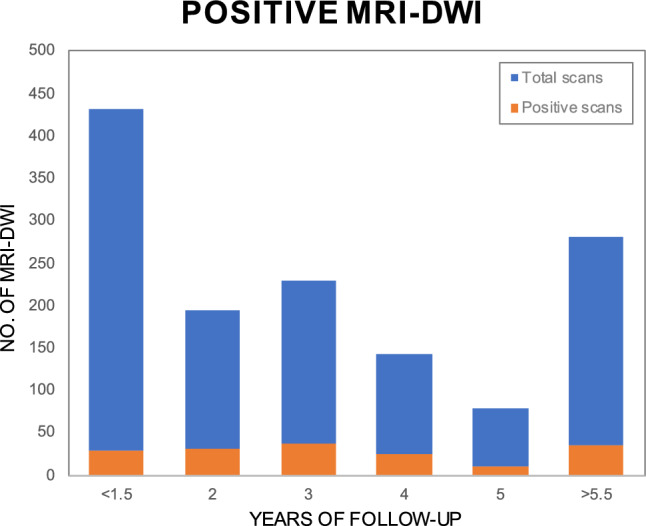
Bar chart showing the number of MRI-DWI obtained per period, including number of positive scans, reflecting the standard FU scheme of approximately 1, 3 and > 5.5 yrs post-operatively. MRI-DWI of known disease were excluded

Most MRI-DWI were obtained < 1.5 yrs of surgery, followed by > 5.5 yrs and 3 yrs post-operatively, reflecting the standard FU scheme in our centre (Fig. 2).

**Fig. 3:**
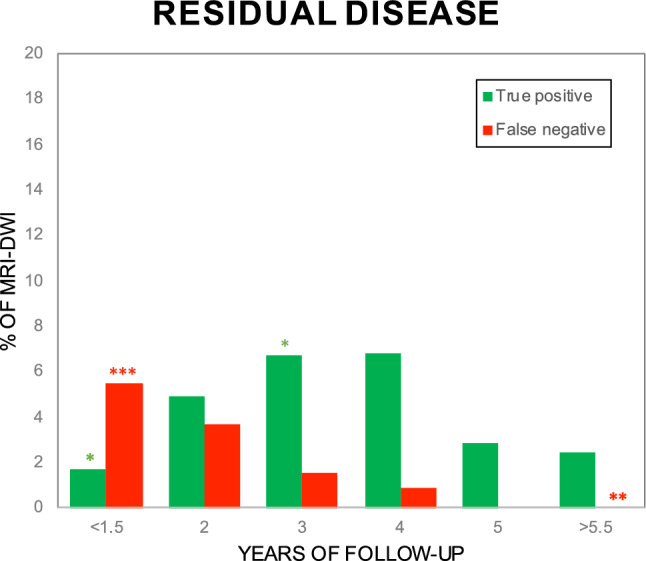
Bar chart showing number of false negative and true positive scans, as percentage of total MRI-DWI obtained. The highest rate of false negative scans was found < 1.5 yrs; the highest rate of true positive scans at approximately 3 yrs after initial surgery, compared to other FU periods. MRI-DWI of known disease were excluded. Please note maximum of y-axis is 20%. *p < 0.05, **p < 0.01 and ***p < 0.001

Within 1.5 yrs, more FN MRI-DWI were found (22/65, *p* < 0.001) than in the other FU periods, while less TP MRI-DWI were found (7/65, *p* < 0.05) (Fig. 3 and supplemental table 1). At approximately 3 yrs of FU, a higher proportion of verified scans were found to be TP than in other periods (13/25, *p* < 0.05). The latest TP MR-DWI was detected at 7.5 yrs after initial transcanal procedure, following two FN MRI-DWI at 1.2 and 4.3 yrs.

**Table 2:** Characteristics of 664 initial surgeries performed

Surgery *n* = *664*	n (%)
Canal wall up mastoidectomy w/obliteration	289 (43.5%)
Canal wall up mastoidectomy	210 (31.6%)
Transcanal procedure	67 (10.1%)
Reconstruction of the posterior canal wall w/obliteration	36 (5.4%)
Subtotal petrosectomy	24 (3.6%)
Canal wall down mastoidectomy	23 (3.5%)
Canal wall down mastoidectomy w/reconstruction of the posterior canal wall and obliteration	15 (2.3%)

### Diagnostic parameters

**Fig. 4:**
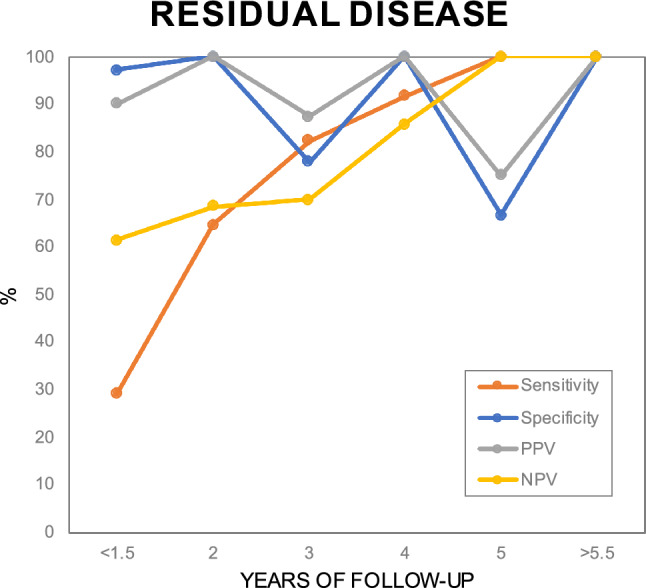
Scatterplot illustrating an increase of sensitivity and NPV of MRI-DWI in detecting residual disease over length of FU, while specificity and PPV fluctuate but remain relatively high. *PPV* positive predictive value, *NPV* negative predictive value

The sensitivity of MRI-DWI in detecting residual disease was 65.2% overall, passing 80% from approximately 3 yrs of FU onwards (Fig. 4). Specificity of MRI-DWI reached 94.6% overall. PPV was higher than NPV (93.8% vs 68.6%). Within 1.5 yrs sensitivity and NPV were relatively low, while both diagnostic parameters increased with duration of FU.

### Surgery type

The majority of TP MRI-DWI were obtained after surgery without obliteration (34/44, *p* < 0.001). Obliteration did not lead to a significantly higher rate of FP MRI-DWI. Most FN MRI-DWI were found after surgery without obliteration (22/32, *p* < 0.001). All FN MRI-DWI (n = 32) were found after surgery with an intact canal wall or after trans-canal procedures. Surgery type did not influence time of manifestation.

### Age

A lower age was correlated with a higher rate of TP MRI-DWI (*p* < 0.05). Sub analyses showed patients < 12 yrs were associated with more TP MRI-DWI than patients > 12 yrs (12/189 vs 32/1003, *p* < 0.05*).* This specific group also had a higher rate of FN MRI-DWI (10/189 vs 22/1003, *p* < 0.05). No significant difference in time of manifestation was noted between adults and patients < 12 yrs.

## Discussion

### Strengths of this study

This is, to the best of our knowledge, the largest retrospective study to date analysing the accuracy of MRI-DWI in detecting residual cholesteatoma across multiple surgery types with a generally long FU. Scan outcomes were verified per-operatively and specifically residual disease was noted (as oppose to recidivism in general), increasing accuracy of diagnostic parameters. As this study was done retrospectively, it is a realistic evaluation of the applicability of MRI-DWI in detecting residual cholesteatoma in clinical practice.

### MRI-DWI findings

Our study revealed a substantial amount of FN and FP MRI-DWI in radiologic FU. This underlines the importance of correlating findings of MRI-DWI with per-operative findings, rather than considering accuracy of imaging based on clinical FU. Causes of FP MRI-DWI findings are in line with other studies, reporting a Silastic™ sheet or accumulated ear wax against a retracted tympanic membrane to potentially interfere with cholesteatoma detection [23, 28, 29]. In our centre, ear wax removal at the outpatient clinic before obtaining MRI-DWI is done regularly and could be considered standard preparation.

### Diagnostic parameters

Our reported rate of MRI-DWI sensitivity in detecting residual disease is in line with literature [5, 7, 10, 13–24]. Compared to a recent meta-analysis of MRI-DWI sensitivity and specificity in detecting recidivism (respectively 93% and 91%) [10], our rates are slightly lower. In most studies, MRI-DWI is performed on basis of clinical suspicion of residual disease [14, 15, 17, 19, 22, 30], resulting in a higher incidence of residual cholesteatoma and introducing a selection bias. We expect reduced selection bias in our data, as the scans in our cohort were performed in all patients as part of routine FU besides clinical suspicion. Moreover, scans are often scored for study purposes [13, 22, 24], improving diagnostic parameters. In this study, scans were read by a group of head- and neck and neuroradiologists with different levels of experience as part of normal clinical routine. Therefore, our results give a more realistic representation of the diagnostic value of MRI-DWI in routine daily practice with a higher external validity.

Even though 1.5T systems seem to be more common in the current literature, the use of 3.0T compared to 1.5T for radiologic FU of cholesteatoma has shown to be indifferent [31]. Furthermore, there were no evident differences in diagnostic parameters before and after the alteration of b-value used.

### Diagnostic parameters stratified by period of FU

This study illustrates a greatly varying prognostic value of MRI-DWI over length of FU, undoubtedly contributing to the wide range of reported diagnostic parameters. The benefit of early scanning can be questioned by the limited diagnostic accuracy within 1.5 yrs of surgery. Previous studies report current MRI-DWI are able to detect cholesteatoma at 2–3 mm and suggest radiological diagnosis of residual cholesteatoma is possible at 9–12 months [10]. However, growth rate of residual cholesteatoma varies [32, 33] and therefore imaging obtained rather shortly after primary surgery can lead to a considerable amount of FN. After approximately 3 yrs of FU, sensitivity of MRI-DWI for residual disease surpasses 80% and significantly more TP MRI-DWI were obtained. This is in line with a recent study reporting an average interval between initial surgery and positive MRI-DWI of 3.8 yrs (of which almost all positive scans were confirmed to be cholesteatoma per-operatively) [32].

### Surgery type

This study confirms the role of obliteration in reducing residual disease [25, 33–36]. Multiple theories could explain the latter: prior to obliteration, complete visualization of the anterior epitympanic space is crucial. This could expose missed disease, as this space is prone to residual disease [37, 38]. Removal of the malleus head and incus reduces the risk of leaving cholesteatoma on an intact ossicular chain [39]. Potentially obliteration could terminate proliferation of any residual epithelial cells (“Hinohira effect” [40]). Lastly, the surgeon could be even more vigorous, as revision surgery after obliteration will not be simple.

Obliteration was not associated with more FP MRI-DWI, perhaps due to the obliteration material used (hydroxyapatite granules). Similar to the “Hinohira effect”, obliteration potentially prevents the formation of granulation tissue or cholesterol granuloma in the mastoid cavity, two frequent causes of FP findings [15, 23]. Also, obliteration of the mastoid cavity was associated with less FN MRI-DWI. This could be due to growth of epithelial remnants in a compressed space, facilitating the threshold of detection with MRI-DWI. Residual disease was not found earlier after obliteration and therefore a shortened interval between initial surgery and first MRI-DWI in obliterated ears is not necessary.

Patients who underwent canal wall up surgery had a higher chance of FN MRI-DWI, demanding increased awareness after a negative scan.

### Age

Our study confirmed a higher risk of residual disease in cholesteatoma acquired at a young age [25, 35, 41, 42]. MRI-DWI FU is widely applied in the paediatric population [17, 43] and previously it has been suggested to increase frequency of MRI-DWI FU in this group [10, 43]. As we did not find residual cholesteatoma earlier in the paediatric group, shortened intervals between initial surgery and radiologic FU do not seem necessary. As recidivism of paediatric cholesteatoma mostly occurs within 5 yrs [44], a “late” scan to detect delayed residual disease, conform the adult population, is warranted. The higher rate of FN MRI-DWI found in patients < 12 yrs could be due to the specific growth pattern of paediatric cholesteatoma: invasive rather than forming a dense keratin pearl with associated diffusion restriction.

### Optimized FU scheme

The diagnostic accuracy of MRI-DWI for FU of residual cholesteatoma could be increased by optimizing a standardized FU scheme. If correctly applied, MRI-DWI can replace a standard second-look procedure. In a stable ear, the first routine FU scan (an “early” scan) can be obtained approximately 3 yrs post-operatively. If the result is doubtful, we suggest to repeat it after 12 months. A “late” scan can be obtained after approximately 5 yrs. High risk patients, i.e., patients < 12 yrs or patients after surgery with an intact canal wall and without obliteration, show how higher FN MRI-DWI rates. We therefore suggest an “extra late” scan approximately 9 yrs after initial surgery in this high-risk group, in order to catch previously missed disease. Shortened intervals for these specific patients does not seem necessary.

Detecting cholesteatoma approximately 3 yrs after initial surgery might raise the question whether this is not “too late”. The Covid pandemic has raised similar concerns regarding increased delays between cholesteatoma diagnosis and surgery. Increased waiting times up to 1 yr have not been associated with increased recidivism or major complications defined as facial nerve palsy or intracranial complications [45]. It could be argued early detection and subsequent surgery prevent further damage of the ossicular chain. However, the question remains if this would lead to better audiologic outcomes [46, 47]. Furthermore, doubts concerning dubious outcomes of early scans lead to further wait-and-scan FU, wasting costly resources. Starting radiologic FU approximately 3 yrs after initial surgery increases diagnostic accuracy, reduces unnecessary imaging, misdiagnosis and potentially even prevents unwarranted second-look procedures. This reduces costs whilst also keeping sustainability in mind. In our opinion, FU scans within 1.5 yr of surgery should be limited and solely reserved for unstable ears with clinical suspicion of residual disease, warranting early intervention.

**Fig. 5:**
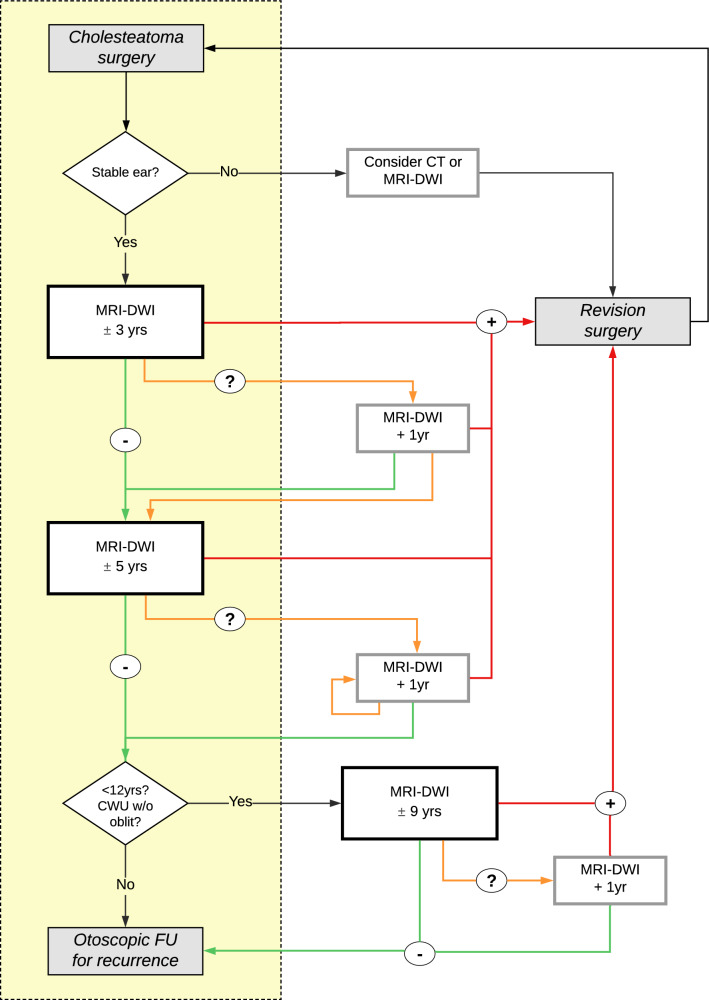
Flowchart of MRI-DWI follow-up scheme after cholesteatoma surgery: standard MRI-DWI after approximately 3 and 5 yrs, as well as an MRI-DWI after approximately 9 yrs in patients with specific risk factors. “− ” indicates no cholesteatoma was identified on MRI-DWI, “?” indicates a dubious result of MRI-DWI and “ + ” indicates a cholesteatoma identified on MRI-DWI. FU: follow-up; w/o: without

The “late” scan is crucial to reveal delayed growth of residual disease. A scan after 4 or 5 yrs has been suggested previously, as up to 31% of MRI-DWI turn positive after initial negative MRI-DWI, without any clinical signs of disease [11, 32]. Also, long-term residual disease is reported up to a decade after initial surgery and some studies therefore suggest lifelong surveillance [32]. We identified specific patient and surgical characteristics that lead to a higher risk of residual disease and a higher risk of FN MRI-DWI, necessitating a longer period of radiologic FU. A flowchart of our suggested optimized FU scheme is provided in Fig. 5.

### Study limitations and suggestions for future research

The retrospective set-up of our study has obvious restrictions, such as the risk of slight selection bias and assessment of imaging by a group of head- and neck and neuroradiologists with different levels of experience. Re-evaluation of FN and FP MRI-DWI by an experienced head- and neck and neuroradiologist yielded 1/4 FP and 4/32 FN MRI-DWI misinterpretations, while 3/4 FP and 12/32 FN MRI-DWI were confirmed. Retrospectively, a focus too small to classify was identified in 8/32 FN MRI-DWI. In another 8/32 FN MRI-DWI (all obtained in 2012–2014), a dubious focus was found. The latter scans were found to be of lesser quality than later scans, most probably due to the use of an older scanner in that period. Recalculations of diagnostic parameters after correction of misinterpreted MRI-DWI did not influence our conclusions significantly.

Furthermore, this study was performed in a tertiary referral centre, potentially influencing type of cholesteatoma and respective surgery performed. An under-coverage bias in our study may result in a slight underestimation of sensitivity and specificity rates; it is expected that a larger proportion of the negative MRI-DWI that are still in FU are true negative rather than false negative. Furthermore, timing of TP MRI-DWI was used to estimate timing of disease manifestation. However, the timing of routine scans followed our standard FU scheme and therefore may not directly reflect the exact timing of disease manifestation. It is also possible incidental FN and FP MRI-DWI were missed in the case of coincidental presence of both FN and FP within one ear, for instance a Silastic™ sheet and a small residual pearl. We are aware of contra-indications of MRI-DWI, such as patients with cochlear implants or severe claustrophobia. This group, however, is expected to be a negligible.

Ideally, a prospective study would obtain MRI-DWI in all patients planned for a second-look procedure in both secondary and tertiary referral centres, based on clinical suspicion or planned routinely at various months to years of FU. In the future, long-term outcomes of reduction of surgical interventions, scans, costs and carbon footprint as well as safety and the occurrence of adverse events in the proposed FU scheme could be analysed. The introduction of fused CT and MRI-DWI, a novelty in our institution, can also further enhance diagnostic accuracy of MRI-DWI FU (supplemental Fig. 1). Also, MRI-DWI FU does not replace out-patient clinic visits for detection of recurrent disease, as a novel retraction pocket can form at any moment after surgery. An optimum FU scheme to detect recurrences was beyond the scope of this study and could be the subject of further research.

## Conclusion

Our study shows that radiologic FU early after surgery poses the risk of FN MRI-DWI. Scanning after approximately 3 yrs yields a significantly higher rate of TP MRI-DWI for residual disease than other FU periods, while sensitivity of the scan in detecting residual disease surpasses 80%. Our novel FU scheme proposes standard MRI-DWI after approximately 3 and 5 yrs, as well as an additional MRI-DWI after approximately 9 yrs in patients with specific risk factors (i.e., < 12 yrs or after canal wall up surgery without obliteration).

## Supplementary Information

Below is the link to the electronic supplementary material.

Supplemental Fig. 1: 1. False negative MRI-DWI – Axial MRI-DWI obtained 372 days after canal wall up surgery due to a cholesteatoma of the left ear, showing no suspicious lesions (A). Two years later a hyperintense lesion was found on axial MRI-DWI (B). The corresponding fused MRI-DWI/CT in coronal plane (C) depicted a suspicious lesion in the epitympanum, which was confirmed to be residual cholesteatoma per-operatively. 2. False positive MRI-DWI – Axial MRI-DWI (A), fused MRI-DWI/CT in axial plane (B) and coronal plane (C) obtained 2 years after canal wall-up procedure with obliteration using hydroxy-appetite granules due to cholesteatoma of the left ear, depicting a hyperintense epitympanic lesion with possible destruction of the middle fossa plate, suggestive of a cholesteatoma. Per-operatively no cholesteatoma was found.

Supplemental table 1: Outcomes of MRI-DWI per period of FU. 

## Data Availability

The authors confirm that the data supporting the findings of this study are available within the article and its supplementary materials.

## Abbreviations

EPI: Echo planar imaging
FU: Follow up
MRI-DWI: Magnetic resonance imaging, diffusion weighted imaging
OCR: Ossicular chain reconstruction
TP: True positive MRI-DWI, i.e., scan marked positive and subsequent surgery revealed residual cholesteatoma
FP: False positive MRI-DWI, i.e., scan marked positive but subsequent surgery showed no presence of disease
TN: True negative MRI-DWI, i.e., scan marked negative and subsequent surgery confirmed no presence of disease
FN: False negative MRI-DWI, i.e. scan marked negative but subsequent surgery showed residual cholesteatoma
PPV: Positive predictive value
NPV: Negative predictive value
