# Supplementary figures and images for: MRI-DWI detection of residual cholesteatoma: moving toward an optimum follow-up scheme
Source: Eur Arch Otorhinolaryngol. 2024 Sep 13;282(2):659–68. doi: 10.1007/s00405-024-08939-9 (PMC11805829; doi:10.1007/s00405-024-08939-9)

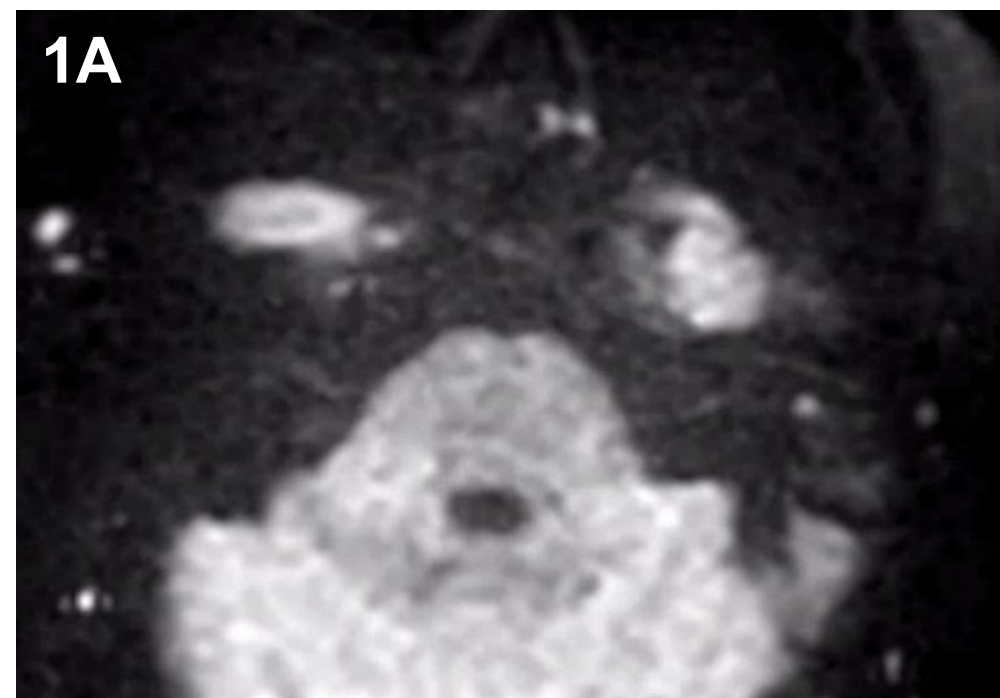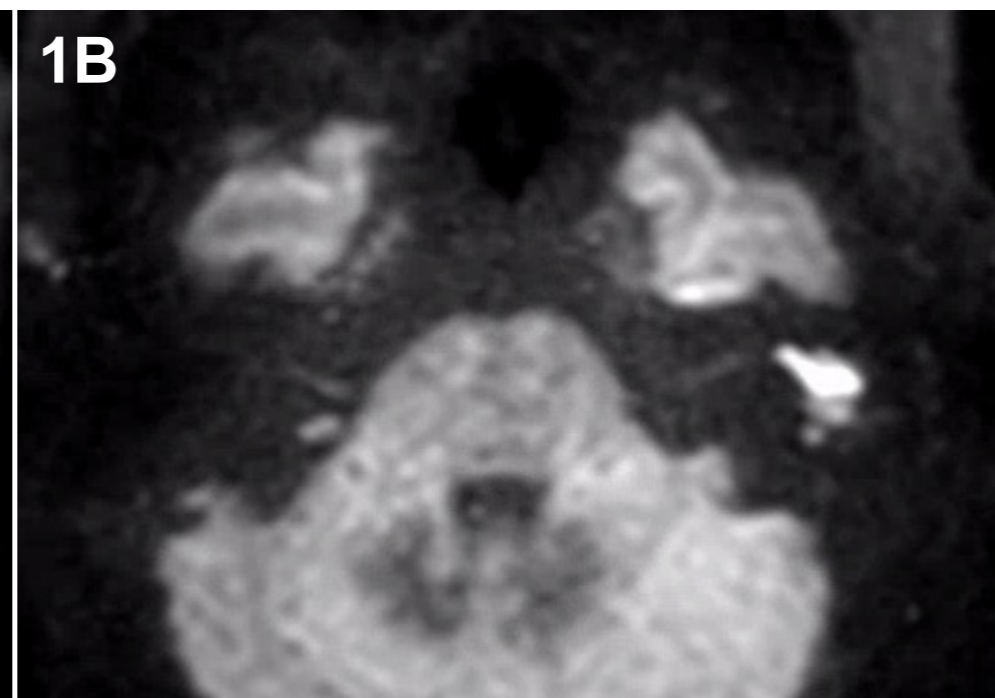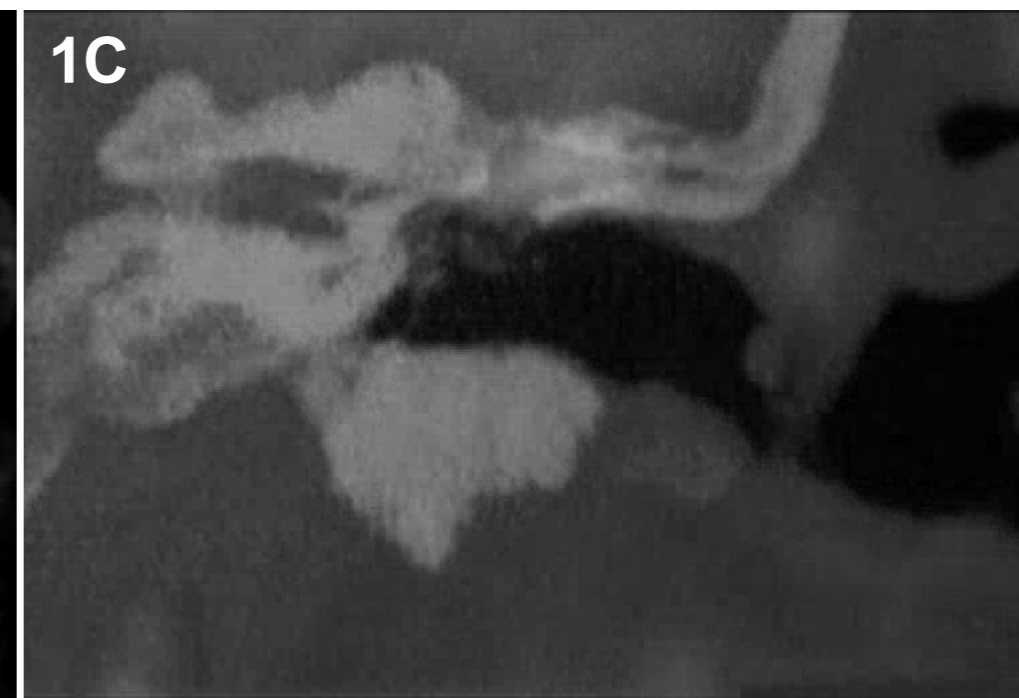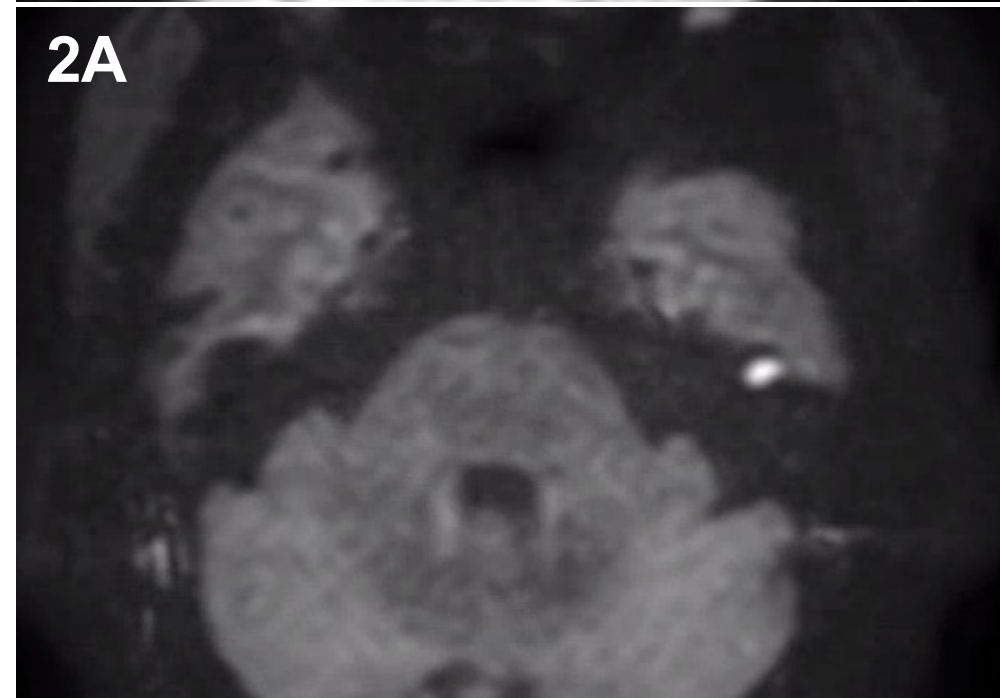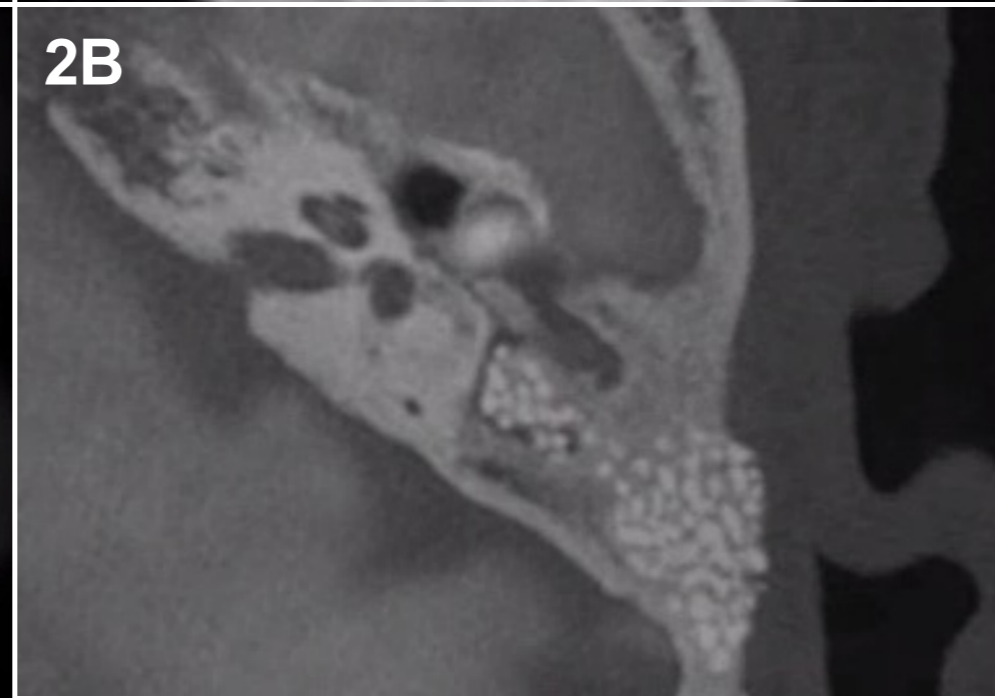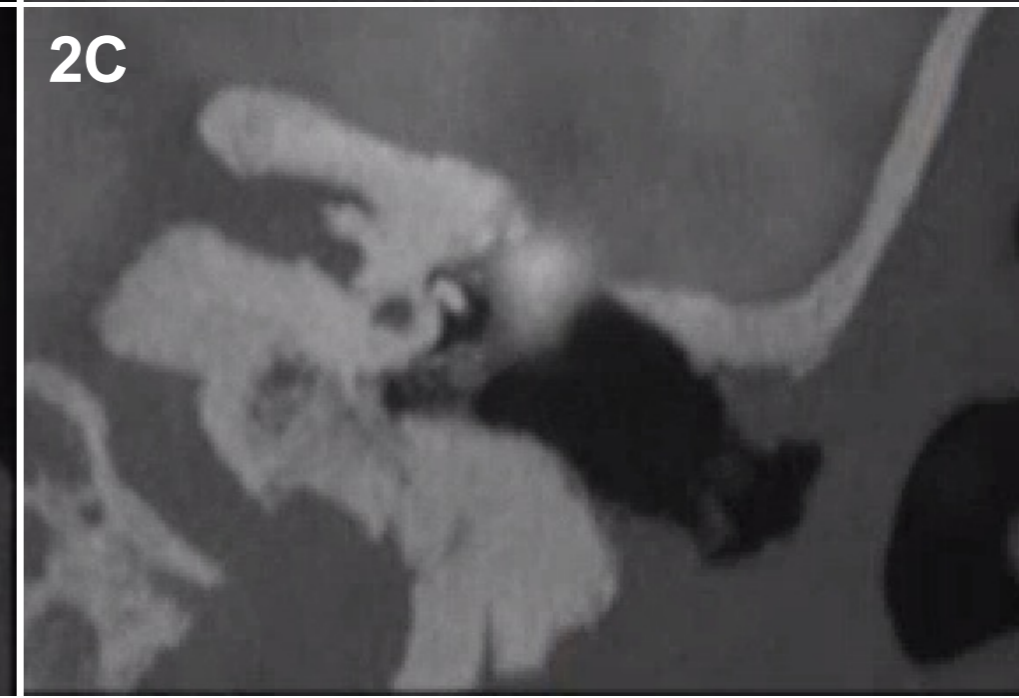

Supplement: Supplementary file 1 — Supplemental Fig. 1: 1. False negative MRI-DWI – Axial MRI-DWI obtained 372 days after canal wall up surgery due to a cholesteatoma of the left ear, showing no suspicious lesions (A). Two years later a hyperintense lesion was found on axial MRI-DWI (B). The corresponding fused MRI-DWI/CT in coronal plane (C) depicted a suspicious lesion in the epitympanum, which was confirmed to be residual cholesteatoma per-operatively. 2. False positive MRI-DWI – Axial MRI-DWI (A), fused MRI-DWI/CT in axial plane (B) and coronal plane (C) obtained 2 years after canal wall-up procedure with obliteration using hydroxy-appetite granules due to cholesteatoma of the left ear, depicting a hyperintense epitympanic lesion with possible destruction of the middle fossa plate, suggestive of a cholesteatoma. Per-operatively no cholesteatoma was found. [file 405_2024_8939_MOESM1_ESM.pdf]
