# Supplementary material for: MRI-DWI detection of residual cholesteatoma: moving toward an optimum follow-up scheme
Source: Eur Arch Otorhinolaryngol. 2024 Sep 13;282(2):659–68. doi: 10.1007/s00405-024-08939-9 (PMC11805829; doi:10.1007/s00405-024-08939-9)
Supplement: Supplementary file 2 — Supplemental table 1: Outcomes of MRI-DWI per period of FU. [file 405_2024_8939_MOESM2_ESM.docx]

Supplemental table 1:

|  |  |  | | | |
| --- | --- | --- | --- | --- | --- |
| **Years of follow-up** | ***MRI-DWI*** *(n)* | | | | |
|  | *True positive* | *True positive*  *excl known disease* | *True negative* | *False positive* | *False negative* |
| <1.5 | 9 | 7 | 35 | 1 | 22 |
| 2 | 11 | 8 | 13 | 0 | 6 |
| 3 | 14 | 13 | 7 | 2 | 3 |
| 4 | 11 | 8 | 6 | 0 | 1 |
| 5 | 3 | 2 | 2 | 1 | 0 |
| >5.5 | 12 | 6 | 7 | 0 | 0 |
| Total | 60 | 44 | 70 | 4 | 32 |
